# Hyaluronan promotes intracellular ROS production and apoptosis in TNFα-stimulated neutrophils

**DOI:** 10.3389/fimmu.2023.1032469

**Published:** 2023-02-06

**Authors:** Iwona Niemietz, Kelly L. Brown

**Affiliations:** ^1^ Department of Microbiology and Immunology, The University of British Columbia, Vancouver, BC, Canada; ^2^ BC Children’s Hospital Research Institute, Vancouver, BC, Canada; ^3^ Department of Pediatrics, The University of British Columbia, Vancouver, BC, Canada

**Keywords:** neutrophils, priming, hyaluronan, tumor necrosis factor-alpha, reactive oxygen species

## Abstract

**Background:**

Hyaluronan (HA) is an important structural component of the extracellular matrix and has well-described roles in maintaining tissue integrity and homeostasis. With inflammation, HA metabolism (synthesis and degradation) increases and results in higher concentrations of soluble HA. Previously, we demonstrated that (soluble) HA primed resting neutrophils for the oxidative burst in response to a secondary stimulus. Notably, HA-mediated priming was not dependent on degranulation, which is a hallmark of priming by classical agents such as TNFα. In this study, we queried the ability of HA to prime neutrophils to different stimuli and its capacity to modulate neutrophil function in the presence of TNFα.

**Methods:**

Blood neutrophils from healthy donors were stimulated *ex vivo* with HA in the absence and presence of classic neutrophil agonists, inclusive of TNFα. Western blotting was used to assess the activation (phosphorylation) of p38 MAPK, and key neutrophil functions associated with priming and activation, such as intracellular and extracellular ROS production, degranulation, and apoptosis, were evaluated by standard chemiluminescence assays (ROS) and flow cytometry.

**Results:**

Hyaluronan is capable of atypical priming and, with TNFα, co-priming neutrophils for an enhanced (rate and/or magnitude) oxidative burst to various secondary stimuli. In addition, HA can augment intracellular ROS production that is directly induced by TNFα in resting neutrophils, which coincided with the activation of p38 MAPK and apoptosis.

**Conclusions:**

These data demonstrate that the extracellular matrix component HA is a key modulator of neutrophil function(s) in the presence of inflammatory agents such as TNFα. Moreover, it provides additional evidence for the diversity and complexity of neutrophil priming and activation during inflammation.

## Introduction

Hyaluronan (HA) is a naturally occurring glycosaminoglycan and a ubiquitous component of the extracellular matrix (ECM) (1). In homeostatic tissue, HA undergoes continuous synthesis and enzymatic degradation that is mediated by hyaluronan synthases and hyaluronidases, respectively. This basal rate of HA synthesis and corresponding degradation is increased during inflammation (2) and to an extent that circulating concentrations of HA are elevated in individuals with conditions characterized by systemic inflammation, such as rheumatoid arthritis (3, 4), myositis (5), and sepsis (6). High circulating concentrations of HA have also been observed in other diseases where damage-induced neutrophil infiltration is prominent, such as pulmonary disease (7–9), liver fibrosis (10), lupus nephritis (11) and vasculitis (12).

It is appreciated that the ECM, and components thereof that are liberated during inflammation, can actively participate in immune processes, often by direct engagement of immune cells (13), and ultimately shape the immune response (14). It has been speculated that HA that is degraded during inflammation (by hyaluronidases as per normal metabolism and/or by microbial enzymes and reactive oxygen species present in damaged tissues) may alert host immune cells. Early investigations to this effect provided evidence that HA may act as a damage-associated molecular pattern (DAMP) that induces cytokine production by engaging immune cells through CD44, the primary receptor for HA (15), or Toll-like receptors TLR2 and TLR4 (16–19). However, mounting evidence by our group (20) and others (21, 22) is to the contrary. We recently provided evidence that hyaluronan contributes to innate immune cell activation and inflammation not by the induction of cytokine production by human peripheral blood cells (23) but *via* priming neutrophils for a robust oxidative burst following secondary exposure to the G-protein coupled receptor agonist and bacterial peptide, N-formylmethionyl-leucyl-phenylalanine (fMLF).

Neutrophils are ‘first responders’ of the immune system and the first immune cells to migrate (from the blood stream) into injured and/or infected tissues. Extravasation is initiated by exogenous or host-derived environmental cues that, coincident with inducing a migratory phenotype, can prime and/or fully activate neutrophils to carry out key immune functions. Some stimuli, notably TNFα, put neutrophils in an alerted, or ‘primed’, state while others (e.g., fMLF) fully activate resting or primed neutrophils. Primed neutrophils have several key features including the ability to readily migrate to damaged tissues, possess heightened functional capacity in response to secondary stimuli, and have a prolonged lifespan (24). Primed neutrophils are commonly and conveniently identified by an altered abundance of adhesion molecules (CD11b and CD62L) on the neutrophil surface and enhanced production of reactive oxygen species (ROS) upon (secondary) activation. Classical priming agents, such as TNFα, impart these changes quickly, often within 10 - 20 minutes, and in large part *via* the mobilization and fusion of intracellular granules with the neutrophil plasma membrane (25), which allows for the deposition of granule-stored molecules (e.g., CD11b and the fMLF receptor FPR1) on the cell surface.

In a previous report by our group (23), we provided the first evidence that HA, in the form of both small (< 40kDa) and large polymers (1.01 – 1.8 MDa), is a neutrophil priming agent. However, and in contrast to TNFα and other classical priming agents that induce a primed neutrophil phenotype characterized by altered cell surface proteins (due to granule mobilization), enhanced ROS release to secondary stimuli, and delayed apoptosis, HA-mediated priming is limited to ROS release and is not dependent on granule mobilization. In this study, we strengthen and extend our previous observation of ‘atypical’ priming of neutrophils by hyaluronan, and we provide new evidence that hyaluronan can co-operate with TNFα to co-prime neutrophils for enhanced ROS release. Further, we demonstrate that HA is a modulator of direct effects (not dependent on secondary stimulation) that are induced in neutrophils by TNFα, specifically, the production of intracellular ROS and apoptosis. Altogether in this study, we demonstrate new, context-specific roles for HA in the inflammatory response, that are independent, but perhaps not unrelated to its role in maintaining tissue homeostasis.

## Methods

### Hyaluronan

Pharmaceutical grade hyaluronan (HA), 15M (1.01 - 1.8 MDa) was purchased from Lifecore Biomedical LLC (Chaska, MN, USA). Stocks were reconstituted in endotoxin-free water (GE Healthcare, Chicago, IL, USA) and confirmed to contain < 0.1 EU of endotoxin with a LAL assay (Pierce LAL Chromogenic Endotoxin Quantitation Kit, ThermoFisher Scientific, Waltham, MA, USA) as per manufacturer’s instructions.

### Isolation of human neutrophils

Venous blood from healthy adult volunteers (Children’s and Women’s Research Ethics Board of the University of British Columbia certificate number H15-00351) was collected in sodium heparin vacutainers (BD Bioscience, San Jose, CA, USA). Polymorphonuclear cells (PMN) were isolated using dextran sedimentation (Sigma-Aldrich, Saint Louis, MO, USA) and Ficoll-Paque gradient centrifugation as described (26). Neutrophils consistently comprised no less than 95% of the PMN population (pocHi 100i*™* Hematology Analyzer, Sysmex Corp., Kobe, Japan), thus PMN are referred to as neutrophils throughout this study. Isolated neutrophils were suspended in complete RPMI (RPMI 1640 with 10% fetal bovine serum (FBS), 2 mM L-glutamine and 1 mM sodium pyruvate; all from ThermoFisher Scientific, Waltham, MA, USA) and used immediately. Alternatively, neutrophils were kept on ice in Krebs-Ringer Glucose buffer (KRG, pH 7.3) containing 1 mM Ca^2+^ for a maximum of 6 h prior to use in downstream experiments. Unless otherwise stated, neutrophils (5 x 10^6^/mL) were incubated at 37°C, 5% CO_2_ in the absence and presence of HA (200 μg/mL) and/or TNFα (10 ng/mL) for 20 min.

### Cell surface expression of CD11b and CD35

Following stimulation with TNFα in the absence and presence of HA, the neutrophils were washed, suspended in PBS/2% FBS/5 mM EDTA and stained 45 min on ice with PE-conjugated mouse anti-human CD11b mAb (1:50; Biolegend; #560914) and/or Alexa Fluor^®^ 647-conjugated mouse anti-human CD35 mAb (1:40; Biolegend; #565329). A minimum of 20,000 events in the neutrophil gate, determined by FCS and SSC, were collected on an Attune NxT flow cytometer.

### NADPH oxidase-derived reactive oxygen species

In this study, we specifically measured ROS generated by the NADPH oxidase using a luminol/isoluminol-amplified chemiluminescence assay as described (27). In brief, after incubation with TNFα in the absence and presence of HA, cells in suspension (1 x 10^5^/well) were transferred to a 96-well white flat bottom plate (Nunc™, 1256602), equilibrated 5 min at 37°C in a 200 μL reaction mixture containing KRG with horseradish peroxidase (4 units/mL, Sigma-Aldrich, Saint Louis, MO, USA) and luminol or (membrane impermeable) isoluminol (10 μg/mL, Sigma, Saint Louis, MO, USA) for detection of total ROS and extracellular ROS (ecROS), respectively. To detect intracellular ROS (icROS), membrane impermeable antioxidants, superoxide dismutase (50 units/mL, Sigma, Saint Louis, MO, USA) and catalase (1,000 units/mL, Worthington Biochemical Corp., Lakewood, NJ, USA) were used in combination with luminol. Cells (1 x 10^5^/well) suspended in the reaction mixture were exposed to N-Formylmethionyl-leucyl-phenylalanine (fMLF;100 nM; Sigma, Saint Louis, MO, USA), C5a (50 nM; PeproTech, Rocky Hill, NJ, USA; #300-70), wheat-germ agglutinin (WGA; 10 μg/mL; EY laboratories, San Mateo, CA, USA; #L-2101-25), galectin – 3 (20 μg/mL, courtesy of Dr. H. Leffler, Lund University, Sweden) or PMA (5 mM; Sigma-Aldrich) and chemiluminescence was recorded every 6 sec for a minimum of 10 min on a TECAN Infinite 200 Pro plate reader (TECAN Group, Männedorf, Switzerland). Alternatively, cells were stimulated with HA (200 μg/mL) and/or TNFα (10 ng/mL), and incubated directly in the Tecan at 37°C while chemiluminescence was recorded for a minimum of 60 min.

### Apoptosis

Neutrophils (5 x 10^6^ cells/mL) were incubated in the absence and presence of LPS (anti-apoptotic; 100 ng/mL; *In vivo*Gen, *E.coli* 0111:B4, San Diego, CA, USA), anti-CD95/FasL (pro-apoptotic; 10 μg/mL; Invitrogen by ThermoFisher Scientific, Waltham, MA, USA; #16-0958-81), HA (200 μg/mL) and/or TNFα (10 ng/mL; R&D Systems) for 6 or 20 h at 37°C, 5% CO_2_. Cells were stained sequentially with FITC-Annexin V (1:20, 10 min, BD Bioscience, San Jose, CA, USA) and 7-amino-actinomycin D (7-AAD; 1:100, 5 min, Biolegend, San Diego, CA, USA). A minimum of 50,000 events were acquired on an Attune NxT flow cytometer (ThermoFisher Scientific, Waltham, MA, USA).

### Western blot

Neutrophil lysates were prepared according to the method of Sarkar et al. (28). Briefly, following stimulation, neutrophils (2 x 10^6^ cells) were spun at 350 x *g* for 10 min at 4°C. The cell pellet was lysed in 10% ice cold trichloroacetic acid (TCA) and kept on ice for 5 min. Precipitates were spun at 14, 000 x *g* for 5 min at 4°C. The cell pellet was washed twice in ice-cold acetone (14 000 x g/5min), left to dry for 1 minute at 100°C to ensure residual acetone evaporation, and suspended in 1 x Laemmli buffer. Samples were boiled for 5 min at 100°C and stored at -80°C until use. Lysates (an equivalent of 0.45 x 10^6^ cells/lane) were separated by 10% SDS-PAGE and transferred onto polyvinylidene difluoride membranes (Immobilon-FL, MilliporeSigma). Membranes were blocked with 5% BSA (Sigma) in TBS-T (Tris-buffered saline containing 0.05% Tween-20) for 60 min at room temperature and incubated with primary antibodies overnight at 4°C. Membranes were washed in TBS-T (3 x 5 min) prior to staining with secondary antibodies. After staining with secondary antibodies for 60 min at room temperature, membranes were washed and imaged using a LI-COR Odyssey infrared imager (LI-COR Biosciences). The primary antibodies used were the following: rabbit anti-human p38 mAb (1:2,000; Cell Signalling Technology; #8690), mouse anti-human phospho-p38 mAb (Thr180/Tyr182; 1:2,000; Cell Signalling Technology; #9216). Secondary antibodies used: IRDye 800-conjugated goat anti-rabbit IgG monoclonal antibody (1:20,000; LI-COR; #926-32211; Lincoln, NE, USA) and IgG IRDye 680RD-conjugated goat anti-mouse monoclonal antibody (1:20,000; LI-COR; #926-68070).

### Data analysis

All flow cytometry data were analyzed using FlowJo software (version 10.5, Tree Star Inc, OR, USA). Western blot data were analyzed using Image Studio Lite Quantification Software (LI-COR Biosciences). All data are expressed as mean + standard deviation (SD). Statistical analysis was performed using Graph Pad Prism (version 8.1.2 for Mac OS X, GraphPad Software, La Jolla, CA, USA) on raw data values, as described in each figure legend. Statistical significance is indicated by *p < 0.05, **p < 0.01, ***p< 0.001 and ****p< 0.0001.

## Results

### Hyaluronan primes neutrophils towards select secondary stimuli

We have demonstrated that hyaluronan can prime neutrophils towards secondary stimulation with the exogenous G-protein coupled receptor (GPCR) agonist fMLF (23), resulting in an increased release of extracellular ROS that, on average, was 50-60% greater than induced by fMLF alone (23). Although our prior investigations exclusively used fMLF as the ROS-inducing agent, heightened/amplified ROS production by primed neutrophils can be induced by a variety of both endogenous (e.g., C5a, galectin-3, GM-CSF) and exogenous (e.g., fMLF, LPS, PMA) stimuli (29). Therefore, we asked whether neutrophil responsiveness to ROS-inducing agonists other than fMLF can be primed by HA. Towards this, we evaluated a subset of well characterized agonists that induce ROS production in neutrophils through GPCR (C5a) and non-GPCR mediated signaling (lectins and PMA) and with known differences in the magnitude and kinetics of ROS release.

**Figure 1:**
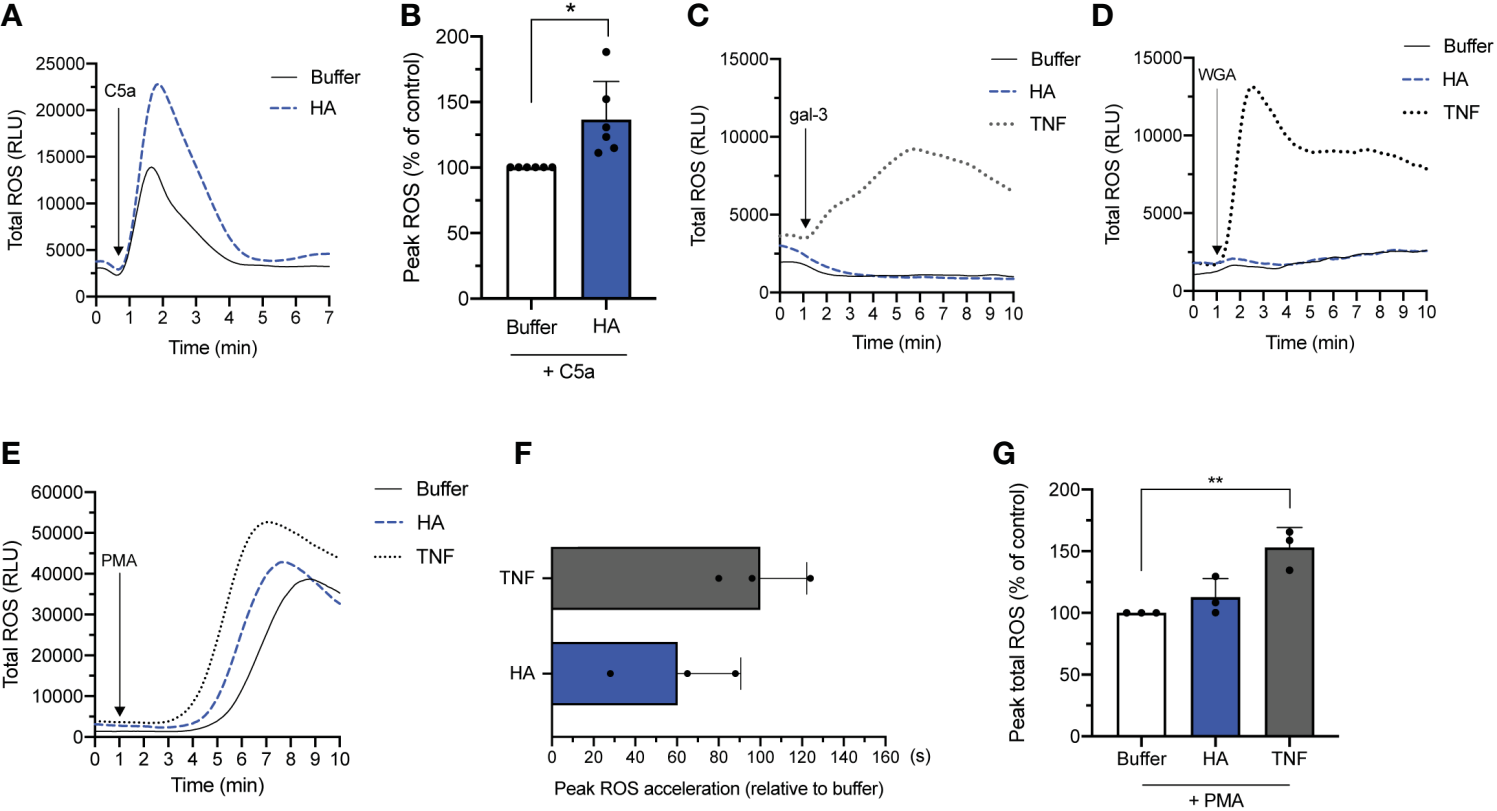
NADPH oxidase-derived ROS release by HA-primed neutrophils activated with various stimuli. Total ROS production (y-axis, relative light units (RLU)) by neutrophils over time (x-axis, min) after **(A)** priming without (buffer) or with HA (200 μg/mL) followed by addition of C5a (50 nM). Data is representative of 6 independent experiments. **(B)** Bar graphs show total ROS production (y-axis) of unstimulated (buffer) and HA-primed cells (HA) after stimulation with C5a (50 nM). The data is presented as mean percentage of peak total ROS (% ROS) + SD normalized to the response of unstimulated neutrophils to C5a, n = 6. **(C)**. Total ROS release (y-axis, RLU) by neutrophils over time (x-axis, min) after priming in the absence (buffer) or presence of TNFα (10 ng/mL) or HA (200 μg/mL) followed by galectin-3 (20 μg/mL) or **(D)** WGA (wheat germ agglutinin; 10 μg/mL). Data is representative of 2 independent experiments. **(E)** Total ROS release (y-axis, RLU) by neutrophils over time (x-axis, min) after priming in the absence (buffer) or presence of TNFα (10 ng/mL) or HA (200 μg/mL) followed by PMA (5 nM). Data representative of 3 independent experiments. **(F)** Bar graphs show the acceleration in reaching peak ROS (x-axis, seconds; mean + SD) by cells primed with TNFα or HA relative to unprimed cells after PMA activation. **(G)** Bar graphs show total ROS production of unstimulated (buffer) and HA-primed cells (x-axis) after stimulation with PMA (5 nM). The data is presented as mean percentage of peak total ROS (% ROS) + SD normalized to the response of unstimulated neutrophils to PMA, n = 3. Statistical analysis was performed using two-tailed paired t-test **(B)** or repeated measures one-way ANOVA followed by Dunnett’s multiple comparisons test, **(G)**; *p < 0.05, **p < 0.01.

In line with the observed response of HA-primed neutrophils to fMLF, HA priming led to a heightened production of ROS by neutrophils subsequently exposed to the endogenous GPCR agonist, C5a. Relative to unprimed cells stimulated with C5a (100% peak ROS release), the average peak ROS release in HA-primed/C5a-stimulated cells was 136.6 ± 29.1% (**Figures 1A, B**).

Next, we evaluated the ability of HA to prime neutrophils for secondary stimulation with non-GPCR agonists. First, we tested the ability of HA to prime neutrophils towards galectin-3 and wheat germ agglutinin (WGA), which are lectins that crosslink cell surface glycoprotein receptors. Contrary to fMLF and C5a that can induce ROS production in resting and primed neutrophils, WGA and galectin-3 (30, 31) can only induce ROS release by classically primed neutrophils by virtue of granule mobilization that results in the deposition of cognate granule-stored receptors on the cell surface (32). In addition, lectin-induced release of ROS by primed neutrophils is lower in magnitude but longer in duration compared to fMLF and C5a; the kinetics and magnitude are shown in **Figures 1C, D** for TNF-α-primed neutrophils. Consistent with the inability of HA to induce neutrophil degranulation, neither galectin-3 nor WGA could provoke ROS release by neutrophils primed with HA.

Finally, we evaluated the responsiveness of HA-primed neutrophils to the diacylglycerol mimetic, PMA, which induces ROS production through a receptor-independent mechanism (33). PMA is a potent ROS-inducing stimulus that alone can induce an oxidative burst equivalent or greater in magnitude to primed/activated neutrophils. In this scenario, priming prior to secondary stimulation with PMA alters (accelerates) the kinetics of the oxidative burst more so than the magnitude; this heightened and hastened ROS production by TNFα-primed neutrophils in response to PMA is shown in **Figure 1E** (34). Like TNFα, HA-mediated priming also shortened the response time (i.e., time to peak ROS release) induced by PMA when compared to buffer. Neutrophils primed with HA reached peak ROS release considerably faster (60.0 ± 30.3 s) than unprimed cells (buffer) exposed to PMA (**Figure 1F**). By way of comparison, TNFα-primed/PMA-activated neutrophils reached peak ROS release 100.0 ± 22.2 sec before unprimed cells. The shortened ROS response time in HA-primed cells was accompanied by a (non-significant) increase in total ROS release (112.8 ± 15.1% of peak total ROS) that was substantially lower in magnitude than in TNFα-primed neutrophils (153.0 ± 16.3% of peak ROS) following PMA activation compared to unprimed neutrophils (buffer; 100% peak ROS release) (**Figure 1G**).

In summary, HA atypically primes neutrophils to respond to GPCR- and non-GPCR stimuli (C5a, PMA and fMLF) with the exception of stimuli (e.g., galectin-3, WGA) that are dependent on granule mobilization (of granule-stored receptors) to exert their ROS-inducing effect (32, 35). Notably, priming induced by HA could modify both the rate and/or magnitude of the oxidative burst.

### Hyaluronan and TNFα co-prime neutrophils for heightened ROS-release

**Figure 2:**
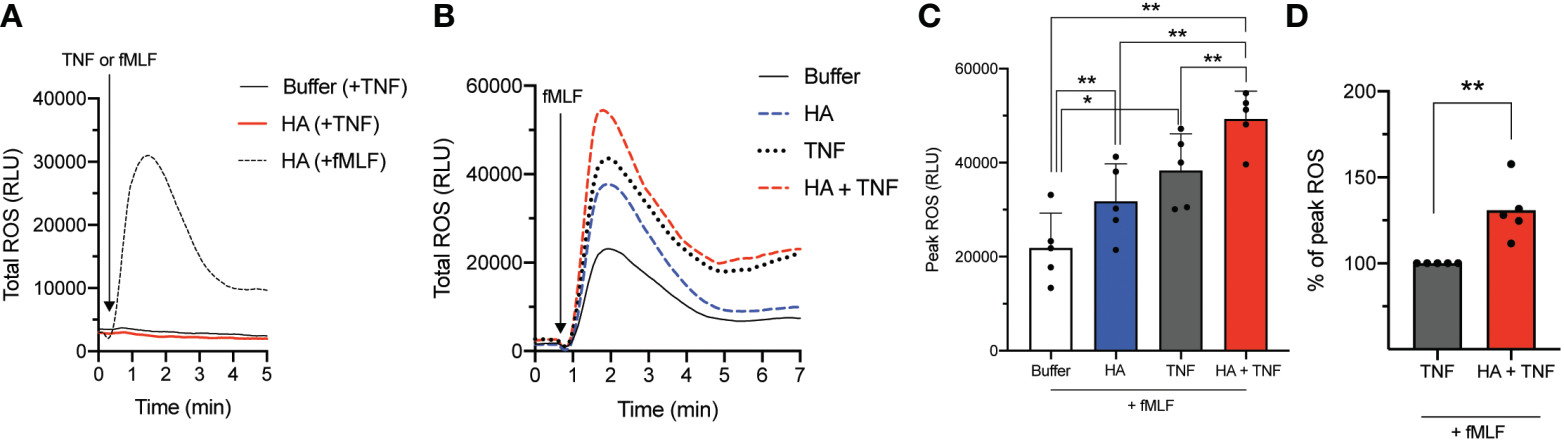
NADPH oxidase-derived reactive oxygen species (ROS) release by co-primed neutrophils. **(A)** Total ROS release (y-axis, relative light units (RLU)) by neutrophils over time (x-axis, min) after priming in the absence (buffer) or presence of HA (200 μg/mL) followed by fMLF (100 nM) or TNFα (10 ng/mL). **(B)** Total ROS production (y-axis, RLU) by neutrophils over time (x-axis, min) after priming without (buffer) and with TNFα (10 ng/mL), HA (200 μg/ml), or HA with TNFα followed by the addition of fMLF (100 nM). Histogram is representative of 5 independent experiments. **(C)** Bar graphs show mean peak total ROS (+SD) for HA-, TNFα−, or HA+TNFα-primed cells (x-axis, RLU) after stimulation with fMLF **(D)** Peak total ROS (mean + SD) in co-primed neutrophils (HA + TNFα) is presented as % of peak total ROS relative to the response of TNFα-primed neutrophils to fMLF, n = 5. Statistical analysis was performed using repeated measures one-way ANOVA with Sidak’s multiple comparison test **(C, D)**; *p < 0.05, **p < 0.01.

Our previous data demonstrated that TNFα and HA elicited priming through overlapping but not identical mechanisms (23). One important difference is the induction of granule mobilization (and the potential deposition of ROS-inducing agonist receptors on the cell surface) by TNFα but not by HA. Our results show that TNFα was not able to induce an oxidative burst in unprimed or HA-primed neutrophils (**Figure 2A**). However, given the different mechanism(s) of priming (34, 36), we questioned if HA- and TNFα-mediated priming was synergistic. To do this, we measured fMLF-induced (total) ROS production by neutrophils primed (20 min) simultaneously with HA and TNFα. Results show that the kinetics of total ROS release was similar in HA-, TNFα-, and HA/TNFα-primed cells (**Figure 2B**). Yet, co-priming of neutrophils with TNFα and HA led to a heightened ROS response to subsequent fMLF activation (peak total ROS = 130.8 ± 16.9% of TNFα-primed/fMLF induced peak total ROS) than with TNFα priming alone (**Figures 2B–D**).

**Figure 3:**
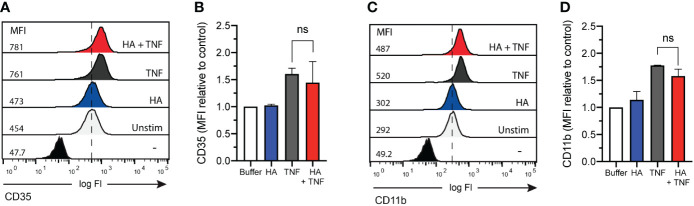
CD35 and CD11b surface expression on co-primed neutrophils. **(A)** CD35 and **(C)** CD11b surface expression (x-axis; log fluorescence intensity (log Fl)) on neutrophils (y-axis; cell counts) following incubation in the absence (unstimulated without (-) and with (Unstim) antibody staining) and presence of HA (200 μg/mL), TNFα (10 ng/mL), or TNFα and HA. Data is representative of 3 experiments. Bar graphs show mean **(B)** CD35 and **(D)** CD11b fluorescence (y-axis; + SD) for no stimulation, HA, TNFα, and HA + TNFα normalized to control (buffer, unstimulated). Statistical analysis was performed using ordinary one-way ANOVA followed by Sidak's multiple comparison test, **(B, D)**; ns - not significant.

Despite the inability of HA to induce granule exocytosis on its own (23), we questioned if granule mobilization was induced in neutrophils co-primed with TNFα and HA. Using flow cytometry, we measured CD35 and CD11b cell surface expression. Although changes in the abundance of CD11b is widely accepted and commonly utilized as a marker of neutrophil degranulation, inclusion of CD35 allows for the assessment of both gelatinase granule and secretory vesicle mobilization. This is important given that the storage of FPR1, the main receptor for fMLF, is predominantly in gelatinase vesicles (that contain CD11b and CD35), but it has also been detected in secretory vesicles (that contain CD35 mostly) (37) that are readily mobilized prior to the degranulation of gelatinase granules (37). In response to TNFα, there was a measured upregulation (mean fluorescence intensity, MFI) of cell surface CD35 and CD11b (**Figures 3A, B** for CD35 and **Figures 3C, D** for CD11b). The addition of HA to TNFα-stimulated neutrophils did not result in the enhanced expression of CD35 or CD11b, suggesting no additional (HA-mediated) mobilization of intracellular granules.

### Hyaluronan enhances intracellular ROS production and apoptosis in neutrophils in the presence of TNFα

**Figure 4:**
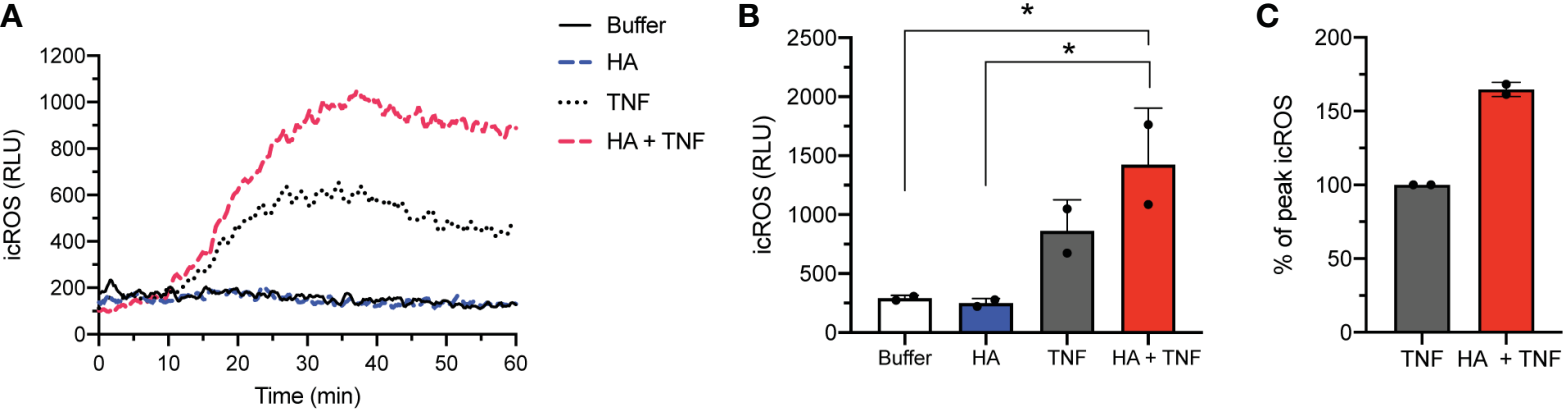
Intracellular ROS release in co-stimulated neutrophils. **(A)** Intracellular ROS production (y-axis, relative light units (RLU)) by neutrophils over time (x-axis, min) after stimulation without (buffer) and with TNFα (10 ng/mL), HA (200 μg/ml), or HA with TNFα. Histogram is representative of 2 independent experiments. **(B)** Bar graphs show mean peak intracellular ROS (icROS, y-axis, RLU) + SD for unstimulated (buffer) or HA-, TNFα−, and HA+TNFα-stimulated cells (x-axis). **(C)** Peak icROS (mean + SD) in co-stimulated (HA + TNFα) neutrophils is presented as % of peak icROS relative to the response of TNFα-stimulated neutrophils, n = 2. Statistical analysis was performed using ordinary one-way ANOVA with Tukey’s multiple comparison test **(B)**; *p < 0.05.

Although priming (by TNFα or HA) and co-priming (TNFα + HA) of neutrophils paired with fMLF activation led to an oxidative burst that was predominantly extracellular (23), some evidence suggests that TNFα can directly stimulate neutrophils to produce intracellular ROS (38, 39). Consistent with this, we observed substantially more intracellular ROS in neutrophils exposed to TNFα alone than cells incubated with HA or no stimulation at all (**Figure 4A**). The addition of HA significantly increased this intracellular, but not extracellular (data not shown), ROS production in TNFα-stimulated cells (**Figures 4A–C**).

Previous reports have demonstrated that modest levels of intracellular ROS that are induced by TNFα in resting neutrophils (38) can trigger apoptotic pathways and accelerate neutrophil apoptosis (40). Likewise, scavenging of intracellular oxygen species delayed apoptosis in neutrophils from healthy individuals and is consistent with delayed apoptosis of neutrophils in patients with chronic granulomatous disease (CGD) that are deficient in the production of NADPH oxidase-derived ROS (41–43). Our prior work shows that HA alone did not affect neutrophil apoptosis (23). However, based on our findings of enhanced production of intracellular ROS in TNFα−stimulated neutrophils in the presence of HA (**Figures 4A–C**), by extension, we questioned whether HA could modulate direct effects of TNFα on neutrophil longevity.

**Figure 5:**
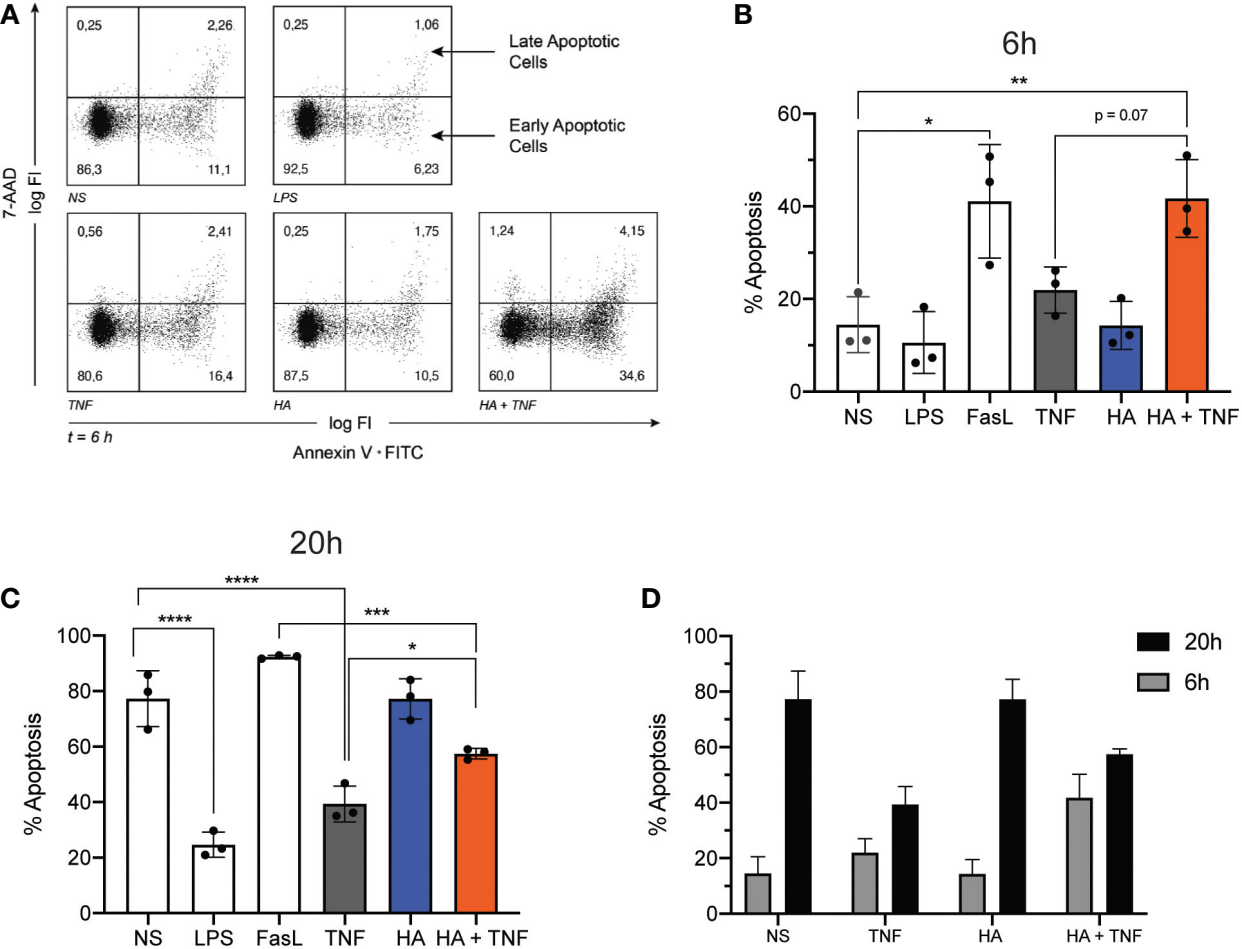
Neutrophil apoptosis in co-stimulated neutrophils. **(A)** Representative dot plots show Annexin V (x-axis; log fluorescence intensity (log FI)) and 7-AAD (y-axis; log FI) staining of neutrophils following ex vivo culture for 6 h (t = 6 h) in the absence of stimuli (NS) or presence of LPS (100 ng/mL), TNFα (10 ng/mL), HA (200 μg/ml), or HA + TNFα. Staining denotes viable (lower left quadrant, Annexin V– 7-AAD–), early apoptotic (lower right quadrant, Annexin V+ 7-AAD–) and late apoptotic (upper right quadrant, Annexin V+ 7-AAD+) neutrophils. Dot plot is representative of 3 independent experiments summarized in B. **(B)** Bar graphs show the mean percent of apoptotic cells (Annexin V+) + SD (y-axis) after 6 h of incubation for no stimulation (NS), LPS (100 ng/mL), FasL (10 μg/mL), TNFα 10 ng/mL, HA (200 μg/mL), and HA + TNFα, n = 3.**(C)** Bar graphs show the mean percent of apoptotic cells (Annexin V+) + SD (y-axis) after 20 h of incubation for FasL (10 μg/mL), LPS (100 ng/mL), TNFα (10 ng/mL), HA (200 μg/mL), and HA + TNFα, n = 3. **(D)** Bar graph shows side by side comparison of the mean percent of apoptotic cells after 6 h (black bars) and 20 h (grey bars) culture, n = 3. Statistical analysis was performed using ordinary one-way ANOVA followed by Tukey’s multiple comparison test, **(B, C)**; *p < 0.05, **p < 0.01, ***p < 0.001, ****p < 0.0001.

To evaluate a potential effect of HA on changes in neutrophil longevity induced by TNFα, the percent of viable, apoptotic, and necrotic neutrophils was determined by AnnexinV/7AAD staining after *ex vivo* culture of neutrophils with HA alone and in combination with TNFα for 6 h and 20 h. At these time points and by an as yet unknown mechanism, TNFα elicits an early (6 h) pro-apoptotic effect on neutrophils and a late (20 h) anti-apoptotic effect (44). After 6 h of neutrophil culture we did not observe a change in the rate of spontaneous apoptosis (sum of Annexin V^+^ 7-AAD^–^ cells/Annexin V^+^ 7-AAD^+^ cells) in cells stimulated with HA (14.3 ± 5.2% apoptotic cells) compared to unstimulated cells (14.5 ± 6.0%) (**Figures 5A, B**). As expected, a greater percent of neutrophils, 21.9 ± 5.0%, were apoptotic after 6 h culture with TNFα alone. This pro-apoptotic effect of TNFα was enhanced (*p* = 0.07) by the presence of HA and resulted in 41.7 ± 8.4% apoptotic neutrophils by 6 h. The percent of apoptotic neutrophils exposed to HA/TNFα was comparable to cells stimulated with FasL, a potent pro-apoptotic stimulus (41.1 ± 12.3%), and significantly higher than measured in unstimulated cells (14.5 ± 6.0%). Expectedly, LPS delayed neutrophil apoptosis, with only 10.6 ± 6.6% apoptotic cells detected after 6 h (**Figures 5A, B**).

Similar to the observed pro-apoptotic effect of HA at 6 h (**Figures 5A, B**), culture of neutrophils for 20 h with TNFα in the presence of HA reversed, at least in part, the anti-apoptotic effect exerted by TNFα alone. In the presence of TNFα, the percent of apoptotic cells was 39.4 ± 6.5% compared to 77.3 ± 10.1% in the absence of stimulation (spontaneous apoptosis). The addition of HA to TNFα-stimulated cells resulted in a significant increase in neutrophil apoptosis (57.5 ± 1.9% apoptotic cells, *p* = 0.03) (**Figure 5C**). Expectedly, neutrophil apoptosis was delayed in the presence of LPS (24.7 ± 4.5% apoptotic cells) and accelerated in the presence of FasL (92.4 ± 0.6%) (**Figure 5C**). HA alone had no effect on neutrophil apoptosis (**Figure 5C**). While confirming our previous results (23) showing that HA was unable to induce apoptosis on its own (summarized in **Figure 5D**), these data suggest a previously undescribed function of HA as a modulator of TNFα-mediated apoptosis.

### Hyaluronan stimulation induces p38 phosphorylation in neutrophils

**Figure 6:**
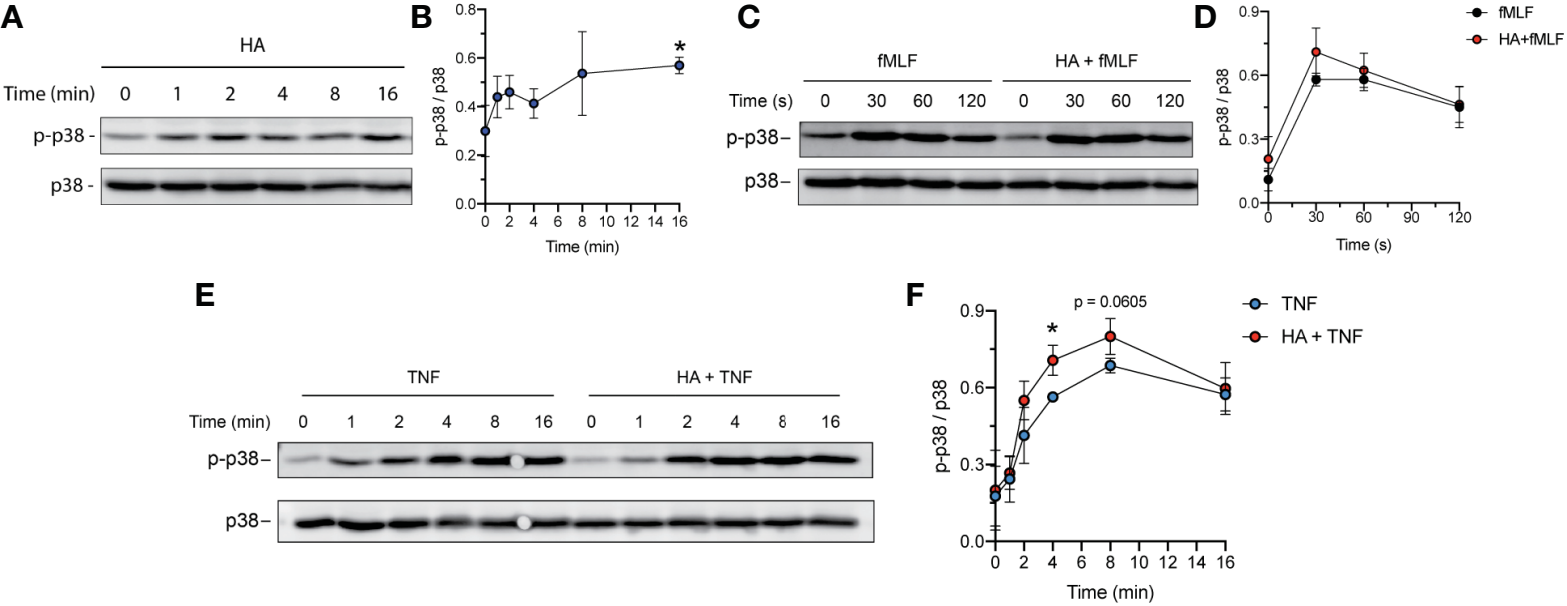
Total and phospho p38 MAPK in TNFα- and HA-stimulated neutrophils. **(A)** Western blot showing the phosphorylated and total p38 MAPK protein over time in neutrophils primed with HA (200 μg/mL). Data is representative of 3 independent experiments. **(B)** Graph shows quantification (mean ± SD) of phospho-p38 normalized to total p38 protein (y-axis) over time (x-axis), n = 3.**(C)** Western blot showing the phosphorylated and total p38 MAPK protein in neutrophils upon fMLF activation (100 nM, 0 – 2 min) at t = 16 min of priming in the presence (HA + fMLF) or absence (fMLF) of HA (200 μg/mL). Data is representative of 3 independent experiments. **(D)** Graph shows quantification (mean ± SD) of phospho-p38 normalized to total p38 protein (y-axis) over time (x-axis) during activation with fMLF at t = 16 min of priming with (HA + fMLF) and without (fMLF) HA, n = 3. **(E)** Abundance of phosphorylated and total p38 MAPK protein over time in neutrophils primed with TNFα (10 ng/mL) and HA + TNFα (200 μg/mL HA). Data is representative of 3 independent experiments. **(F)** Graph shows quantification (mean ± SD) of phospho-p38 normalized to total p38 (y-axis) over time (x-axis) in TNFα-primed and HA+TNFα co-primed neutrophils, n = 3. Statistical analysis for individual time points was performed using unpaired two-tailed t-test, **(B, F)**; *p < 0.05.

The p38 MAPK is well-recognized for its role in cellular stress responses. In neutrophils, rapid phosphorylation of p38 MAPK has been observed during ROS production (45–47) and granule exocytosis (45, 48, 49). p38 MAPK has also been described as a key mediator of apoptosis (50, 51). To investigate the possibility of p38 MAPK activation in response to HA- and/or HA/TNFα stimulation, we assessed phosphorylation of p38 MAPK Thr^180^/Tyr^182^ residues over time using immunoblotting followed by densitometry analysis. Our results show that exposure of neutrophils to HA resulted in rapid phosphorylation of p38 MAPK by ~ 2 min post stimulation and is sustained over the observed 16 min time period (**Figures 6A, B**; 2.1- fold increase in phospho-p38 at 16 min, p = 0.0275). The addition of fMLF, which results in an oxidative burst in HA-primed neutrophils, was associated with a non-significant increase in p38 MAPK phosphorylation, compared to fMLF alone (**Figures 6C, D**).

In contrast to HA, TNFα induced phosphorylation of p38 MAPK had different kinetics, with steadily increasing phosphorylation over a 16-minute time period (**Figure 6E**). Immunoblotting and densitometry analysis of phosphorylated (Thr^180^/Tyr^182^) p38 and total p38 protein in neutrophils co-stimulated (from 0 – 16 min) with TNFα and HA demonstrated more pronounced phosphorylation of p38 (Thr^180^/Tyr^182^) in co-stimulated cells when compared to TNFα alone (**Figure 6E**). Phosphorylation of p38 MAPK in neutrophils exposed to HA/TNFα co-stimulation reached peak activation at ~ 4 min with a maximum fold increase in phosphorylation occurring between 2 - 8 min compared to cells stimulated with TNFα alone (**Figure 6F**). The observed phosphorylation of p38 MAPK in neutrophils exposed HA, as well as enhancement of p38 MAPK phosphorylation in the presence of TNFα, implies that HA-exerted effects are p38 MAPK-mediated.

## Discussion

Building on our previous report of HA as a novel and atypical priming agent for fMLF-induced ROS production by neutrophils (23), herein we demonstrate that HA can prime neutrophils for an augmented oxidative burst in response to classical endogenous and exogenous agonists (receptor and non-receptor mediated) other than fMLF, namely C5a and PMA. HA failed to prime cells for ROS production by lectins, which require an upregulation of granule-stored receptors to exert their ROS-inducing effect (32, 35), thus, providing additional confirmation that HA-mediated priming occurs in the absence of degranulation. Further, HA- and TNFα-induced priming are synergistic,​ leading to an oxidative burst of even greater magnitude that ‘singly-primed’ neutrophils. Co-priming for the neutrophil oxidative burst has been reported previously for TNFα and IFNα (52), but this is the first evidence of neutrophil co-priming with a component of the ECM. The observed synergy between TNFα and HA priming may produce a ‘primed phenotype’ that is found in neutrophils at sites of tissue damage/inflammation where they are in contact with both HA and TNFα simultaneously.

In addition to priming neutrophils, we provide evidence that HA can modulate direct effects of TNFα on neutrophils. Specifically, HA enhances TNFα-induced production of intracellular ROS and exerts a pro-apoptotic effect on neutrophils in the presence of TNFα. Generation of intracellular ROS has been reported to have a role in the transient, pro-apoptotic effect elicited by TNFα in neutrophils (40). Although select stimuli, e.g., GM-CSF, IFNγ, have been reported to enhance ROS production and apoptosis in neutrophils exposed to TNFα (39, 53), again, there is little, if any, prior evidence for a modulatory effect by extracellular matrix components. Few studies have focused on the influence of HA on cell apoptosis in activated cells and studies investigating the effect of HA alone show mixed results (54–57). In leukemic lymphocytes, CD44 stimulation exerted an anti-apoptotic rather than a pro-apoptotic effect (54). Similarly, as reported by Zhao et al., HA delayed apoptosis in murine neutrophils (58). It is important to note that endotoxin contamination of HA (58) was not tested in these studies and cannot be excluded as a driver of delayed apoptosis (59). In agreement with the data presented in this study, a pro-apoptotic effect of HA was observed in lymphoid cells (15), where HA induced cell death in activated but not resting T cells in a CD44-dependent manner.

Our study demonstrates that stimulation of neutrophils with HA is concomitant with the phosphorylation of p38 MAPK. Although the HA-induced activation of p38 was subtle and would benefit from further validation to determine its direct downstream consequences, our data are consistent with evidence that MAPKs, including p38, are active during priming and generation of the oxidative burst (38). It should be also noted that MAPKs have a key role in degranulation (48). However, the extent to which p38 activation contributes directly to NAPDH oxidase activation as opposed to granule mobilization is not known (34, 60). p38 MAPK activation may also regulate ROS generation by promoting cytoskeletal rearrangement that disrupts the interaction of actin with the cytosolic p40^phox^ subunit of the NADPH oxidase (61, 62), and phosphorylation of the NADPH oxidase component p47^phox^. In the context of HA-mediated signaling *via* CD44, Lu et al. (63) demonstrated that medium-sized HA fragments (~ 500 kDa) stimulate p38 MAPK activation in neutrophils and suggest that this is *via* cytoskeletal rearrangements caused by HA binding. It should be noted that p38 MAPK is a mediator of cell apoptosis and at least one study showed that TNFα-induced apoptosis is dependent on the activation of p38 (40). However, as p38 controls ROS production in neutrophils (64) that, in turn, may induce apoptosis (40), it is plausible that the augmented cell death observed in HA/TNFα-stimulated cells is a result of HA-enhanced production of the NADPH-oxidase generated intracellular ROS. This is consistent with an absence of p38 activation in neutrophils stimulated with other pro-, as well as anti-apoptotic stimuli (50). Further analysis is needed to assess whether the enhanced activation of p38 observed in this study results in increased phosphorylation of the p47^phox^ subunit of the NADPH oxidase leading to the observed amplification of ROS generation (65), and if so, whether this is due to independent signals induced separately by HA through CD44-mediated cytoskeletal rearrangement (66) and TNFα engagement and signaling through its cognate receptor (TNFR1).

It is recognized that p38 MAPK phosphorylation may result from TLR4 engagement (67) that may also lead to the activation of NF-κB (68). Demonstrating previously no difference in neutrophil functionality with HA size (< 40kDa up to 1.01 – 1.8kDa polymers) (23), in this study we used the high molecular mass HA polymers that are known to bind to CD44 rather than small HA fragments that may engage CD44 and/or TLR2/4 (summarized by Tavianatou et al. (69)). Accordingly, we found no evidence (based on immunoblot analysis of IκBα degradation) for the activation of the NF-κB pathway in neutrophils incubated with HA (data not shown). This is in agreement with our previous findings (23) showing that HA does not lead to neutrophil responses known to be induced by TLR4 engagement, namely IL-8 release, degranulation and prolonged longevity (70, 71)

Taken together, although HA does not have direct effects on neutrophil activation, our data suggests that is may be a key, context-specific modulator of neutrophil function, namely the production of reactive oxygen species and apoptosis. Considering the interplay between ROS and cell death (72) and in light of the observed modulating effect of HA on TNFα-induced intracellular ROS, the demonstrated pro-apoptotic role of HA is somewhat expected, yet intriguing. Given the potential toxicity of ROS, it is tempting to speculate that by enhancing ROS production, HA contributes to tissue damage. However, by virtue of the pleiotropic roles of both intra- and extra-cellular ROS (73), the consequences of HA-enhanced ROS production are likely more complex. For example, NADPH oxidase-generated ROS may act as a signaling molecule that, among other functions, can regulate cell apoptosis (74). When released into the extracellular space, it can also lead to gap formation between endothelial cells by disturbing junction proteins (75, 76) and inducing cytoskeletal reorganization (77, 78), thereby facilitating cell migration. When considering the pro-apoptotic effect HA had on TNFα stimulated neutrophils, which may be linked to the observed enhancement of TNFα-induced intracellular ROS production, it cannot be excluded that, paradoxically, HA contributes positively to the inflammatory process by facilitating a quick neutrophil response and ensuring a timely termination of inflammation (79); a role that may have pathological consequences when the balance of HA turnover is disturbed and HA is in excess. This could explain the contradicting results where HAS1 (HA synthesizing enzyme) deficiency resulted in chronic inflammation (80), while clearance of hyaluronan with hyaluronidase restored tissue homeostasis (81).

Given that HA is abundant throughout the body, it seems reasonable that HA-mediated effects on neutrophils (the most abundant immune cells) are not direct, but instead governed by the presence of inflammatory stimuli, such as fMLF or TNFα, to ensure that HA-enhanced immune responses are context specific (e.g., upon encountering danger). While an immunomodulatory effect of HA on activated cells has been described in other cells, e.g., T-lymphocytes (15), our findings provide the first report of such a role in neutrophils. HA modulation of TNFα-induced function, particularly the oxidative burst, is intriguing considering that enhanced ROS production at the inflammatory site is commonly thought to contribute to the progression of numerous inflammatory diseases (73), including rheumatic diseases driven by TNFα with concomitant ECM degradation, such as rheumatoid arthritis and vasculitis (82, 83).

In conclusion, our study advances the understanding of the contribution of HA to inflammatory processes that is independent of its previously described role as an inflammation-inducing damage-associated molecular pattern (DAMP) by providing evidence that HA is a modulator of neutrophil effector functions exerted by another stimulus present in the inflammatory milieu, namely TNFα (84).

## Data availability statement

The raw data supporting the conclusions of this article will be made available by the authors, without undue reservation.

## Ethics statement

The studies involving human participants were reviewed and approved by Children’s and Women’s Research Ethics Board of the University of British Columbia. The patients/participants provided their written informed consent to participate in this study.

## Author contributions

IN designed and performed all experiments, prepared figures, and wrote the manuscript. KB oversaw all aspects of experiment design, data analysis, interpretation of results and manuscript preparation. All authors contributed to the article and approved the submitted version.
